# Proposing a new model for location - routing problem of perishable raw material suppliers with using meta-heuristic algorithms

**DOI:** 10.1016/j.heliyon.2019.e03020

**Published:** 2019-12-13

**Authors:** Ali Yaghoubi, Farideh Akrami

**Affiliations:** a Department of Engineering, Raja University, Qazvin, Iran; b Department of Industrial Management, Ghazali Higher Educational Institute, Qazvin, Iran

**Keywords:** Systems engineering, Industrial engineering, Mathematical modeling, Computational intelligence, Process modeling, Industry management, Supply chain management, Ant colony optimization algorithm, Particles swarm optimization algorithm, Perishable raw material

## Abstract

In the last three decades, an integrated approach to optimize logistics system is considered as one of the most important aspects of optimizing supply chain management. This approach involves the ties between locations of facility, allocation of suppliers/customers, structure of transportation routes and inventory control. The aim of this paper is to investigate the ordering planning of a supply chain with multi supplier, multi distribution center, multi customer and one perishable raw material. This paper provides a mathematical model taking in consideration the limitation of raw material corruptibility (perishable material) which belongs to the category of NP-hard problems. To solve the proposed model, the Ant Colony Optimization algorithm (ACO) and Particle Swarm Optimization algorithm (PSO) are employed. In order to improve performances of ACO and PSO parameters, a Taguchi experimental design method was applied to set their proper values. Besides, to evaluate the performance of the proposed model, an example of the dairy industry is analyzed by using MATLAB R 2015a. To validate the proposed meta-heuristic algorithms, the results of them were compared with together. The results of the comparison show that ACO is greater than PSO in speed convergence rate and the number of solutions iterations.

## 1 Introduction

In many logistics environments, decisions should be taken by managers such as locating of distribution centers, allocation of customers to the transportation centers and programing for transportation to provide services for customers. These decisions affect the level of service provided to the customers. The cost of routing and locating affects the entire logistics system. Defining the optimal number and location of distribution centers (warehouses) as well as the schedule of vehicles and distribution routes affect to minimize the total cost of the system. As a result, the mathematical models had been used to determine the location of warehouses and solving these problems. Since many customers can use the same route, it increases the likelihood that its demand will exceed the capacity of the network. This necessity expresses the integration of location and routing problems, but this integration was not recognized until the year 1970.

The location-routing problem can be defined as a routing problem in which determine the locations and the optimum number of warehouses at the same time with vehicles schedules and distribution routes in order to minimize system cost. But most past research in this area has examined the development of location and routing problems separately.

The process of solving the location-routing problem (LRP) consists of three phases: 1. Demand allocation, 2. Facility location and 3. Optimizing vehicle routes of sub-problems can result in an optimized decision. But it's impossible to integrate these problems mathematically. Based on the description provided above, this study presents a new mathematical model taking in consideration the limitation of raw material corruptibility (perishable material) which belongs to the category of NP-hard combinatorial optimization problems. The aim of this paper is to investigate the ordering planning of a supply chain with multi supplier, multi distribution center, multi customer and one perishable raw material. The rest structure of this paper is as follows. Section 2 provides a systematic literature review of the location-routing problem. In Section 3, we present a new multi-objective model for a location-routing problem with considering the limitation of raw material (perishable material) including four objective functions. In section 4, the applications of ACO and PSO algorithms are described to solve the proposed model. The computational analysis is proposed in Section 5. Finally, the conclusions and suggestions are provided in Section 6.

## 2 Literature review

The integration of routing and location problems had not been considered till 1970 and entering the routing problem into the location problem seemed impractical. Baumol and Vinod (1970) attempted to determine the carriers in the unique product market that can decrease the system costs. Constable and Whybark (1978), developed an inventory model with integration of communication costs and demand return rate. This model can minimize communication and inventory costs through determining the inventory re-demand point.

Langley (1979), mixed the communication decisions with inventory location. In this research the schematic plan of facility location decisions, communication and inventory was presented. Van Beek (1981), investigated various strategies for determining the location in a two level distribution system in the production center of a company and considered four local distribution centers. Tuzun and Burke (1999), studied the location–routing modeling with limited capacity of the fleet and presented a super-innovative algorithm base on banned search algorithm that is two-stepped. Ghiani and Improta (2000), presented a new mathematical model based on capable direction location.

Albareda-Sambola et al. (2005), used the web theory for modeling the location – routing problem. Albareda-Sambola et al. (2007), used a new model in order to find a suitable solution to management of dangerous rubbishes. The aim of this research was minimized the system cost and communication risk of treatment and disposal facilities and finding a suitable route to convey dangerous disposals. In order to minimize transportation risks, Alumur and Kara (2007) presented a new model to determine the disposal centers, location of treatment and best routing schedule for various types of hazardous waste.

Vincent et al. (2010), presented Gradual freezing algorithm. To test and validate the proposed algorithm, the classical examples in the literature of the location - routing was used. The results proposed the gradual freezing are superior to other algorithms presented in the literature. In addition to providing new solutions, new hypotheses taken from real life are considered for the location–routing problem. Ngueveu et al. (2010), introduced a model of the router forwarding stacking capacity to minimize the total moving vehicles time. Carter and Ferrin (2011), used a mixture of gradual freezing and branch and bound algorithms in order to solve the problem of location – routing problem considering the hypothesis of delivery of product. Ke and Feng (2013), presented an innovative two-stage method in order to solve the problem of stacked capable communication. Rath and Gutjahr (2014), presented a new optimization model with three-objective functions including: short-term economic, medium-term economic and a disasters objective function. Also, a new meta-heuristic algorithm was designed to solve the proposed model based on genetic algorithm.

Barzinpour and Esmaeili (2014), presented a multi-objective planning model in three levels and in phasic state and used a suitable genetic algorithm to solve it. Agrawal (2014), explored how factories can manage their material sourcing better by expanding suitable raw material sourcing relationships with their suppliers. The results showed that active management of raw material sourcing can add value to supply chains. Marinakis (2015), solved the location–routing problem by using a stacked particles algorithm and a comparison with other algorithms. The results showed that the stacked particles algorithm is a suitable algorithm in location-routing problems. Sattrawut (2015) attempted to reduce the problem solving time through turning the two level necessary materials supply to one level problem. Moreover, they presented a problem with different time horizons and solved it through an accurate algorithm called the branch and price algorithm. Vidović et al. (2016), designed a problem with mixed variables for reuse logistic webs that had two phases and achieved an acceptable result through the innovative algorithm. Azadeh et al. (2017) analyzed inventory decisions and vehicle routing simultaneously. Wu et al. (2017) designed a three location-routing models. They also considered time deadlines and tight time windows to establish services for trains. Hiassat et al. (2017) surveyed location-routing-inventory problem for perishable products distribution. Najjartabar-Bisheh et al. (2018) analyzed the pattern of third-party firms in a tolerable supply chain management. Wang et al. (2018) designed a new LRP model with the minimum total costs as the objective functions, which includes carbon emission costs in cold chain logistics. A new hybrid GA was designed to solve the proposed model.

## 3 Problem description

Methods and techniques of the supply chain are important issues in an organization's supply chain management. The problem was about how to achieve the best and most appropriate method according to the nature of the organization in order to reach the most effective supply chain, always had been one of the biggest challenges in supply discussions. How to provide needed items for an organization is an important issue that will be considered in supply chain management and purchasing. Attention to this fact that which items should be made within the organization and which items should be provided from outside of it, is one of the main issues that had been raised in supply chain management. After determining the most necessary items, the next stage is identifying, choosing and attracting the suitable suppliers in order to provide the needs of the organization. Most of suppliers faced the organizations with different issues about their integration, the manner of communication and specify the other related matters. The chain which has perishable raw materials is more sensitive because of the higher maintenance costs.

On the other hand, the essential parts of the supply chain are routing and transportation process. These processes established material flow between suppliers and customers in an organization. An appropriate distribution system, basically depends on the following parameters: product type, amount of product, size of transportation vehicle and the distance between suppliers to customers.

Based on the description given above, this study proposes a new mathematical model taking in consideration the limitation of raw material corruptibility (perishable material). The aim of this paper is to investigate the ordering planning of a supply chain with multi supplier, multi distribution center, multi customer and one perishable raw material. The most important goals of the proposed model are to minimize transportation costs and transport time of perishable raw materials between suppliers, distribution centers and factory with considering the capacity of them.

In general, the exact algorithms and the meta-heuristic algorithms have been used to solve location-routing problems. The exact algorithms are used for problems with small dimensions and meta-heuristic algorithms for problems with large dimensions. Planning a suitable algorithm, the optimum results can be achieved. Considering the proposed model belongs to the category of NP-hard optimization problems, the ACO algorithm and PSO algorithm are employed to solve it. Also, for improving performances of ACO and PSO parameters, a Taguchi experimental design method is applied to set their proper values.

### 3.1 Model assumption

✓ Each demand center (suppliers and factories) can be assigned to more than one route.
✓ The number of distribution centers and their capacity are limited.
✓ All transportation vehicles are the same in terms of transport capacity and transport speed.
✓ Any transportation vehicles start from a distribution center, and after providing the raw materials of its customers, it will return to the same distribution center.
✓ Multiple uses of vehicles within the routes of the vehicle (time limitation) are possible.
✓ Each of the factories is served only by one vehicle and the vehicle will be back to the start point.

### 3.2 Model formulation

In the following section, the notation, parameters, sets, decision variables of the model and the proposed mathematical model are presented.

#### 3.2.1 Problem parameters

**Table:** 

l	Index of the factories (l = 1,…, L)
i	Index of the distribution center (i = 1,…, I)
j	Index of the supplier centers (j = 1,…, J)
X_ij_^max^	Maximum amount of raw material transported from supplier center j to distribution center i
X_li_^max^	Maximum amount of raw material transported from distribution center i to factory l
D_ij_	Distance from supplier j to distribution center i
D_li_	Distance from distribution center i to factory l
M_i_	Total amount of raw material entered to distribution center i from suppliers
M_l_	Total amount of raw material entered to factory l from distribution centers
U_i_	Maximum capacity at distribution center i
U_l_	Maximum capacity at the factory l
C_i_	Total amount of raw material exported from distribution center i
C_j_	Total amount of raw material exported from supplier j
T^max^	Maximum of the storage time of raw material
α	The rate of transportation cost of raw material per one kilometer
Q_i_	The fixed cost of establishing the distribution center i
Q_l_	The fixed cost of establishing the factory l

#### 3.2.2 Problem variables

**Table:** 

X_ij_	The amount of raw material transported from supplier j to distribution center i
X_li_	The amount of raw material transported from distribution center i to factory l
t_ij_	The required time for transporting raw material from supplier j to distribution center i
t_li_	The required time for transporting raw material from distribution center i to factory l

In terms of the above notation, the new mathematical model taking in consideration the limitation of raw material corruptibility (perishable material) can be formulated as follows:

(1) $$\text{Min}{\text{ ​Z}}_{1}=\overset{\text{I}}{\underset{\text{i}=1}{\sum }}\left(\overset{\text{J}}{\underset{\text{j}=1}{\sum }}{\text{αD}}_{\text{ij}}{\text{X}}_{\text{ij}}{+\text{Q}}_{\text{i}}\right)+\overset{\text{L}}{\underset{\text{l}=1}{\sum }}\left(\overset{\text{I}}{\underset{\text{i}=1}{\sum }}{\text{αD}}_{\text{li}}{\text{X}}_{\text{li}}{+\text{Q}}_{\text{l}}\right)$$

(2) $$\text{Min}{\text{ ​Z}}_{2}=\overset{\text{I}}{\underset{\text{i}=1}{\sum }}\overset{\text{J}}{\underset{\text{j}=1}{\sum }}{\text{t}}_{\text{ij}}{\text{X}}_{\text{ij}}+\overset{\text{L}}{\underset{\text{l}=1}{\sum }}\overset{\text{I}}{\underset{\text{i}=1}{\sum }}{\text{t}}_{\text{li}}{\text{X}}_{\text{li}}$$

Subject to:

(3) $${\text{X}}_{\text{ij}}\leq {\text{X}}_{\text{ij}}^{\text{max}}\;\left(\text{i}\in \text{I, j}\in \text{J}\right)$$

(4) $${\text{X}}_{\text{li}}\leq {\text{X}}_{\text{li}}^{\text{max}}\;\left(\text{i}\in \text{I, l}\in \text{L}\right)$$

(5) $${\text{M}}_{\text{i}}=\overset{\text{J}}{\underset{\text{j}=1}{\sum }}{\text{X}}_{\text{ij}}\;\left(\text{i}\in \text{I}\right)$$

(6) $${\text{M}}_{\text{l}}=\overset{\text{I}}{\underset{\text{i}=1}{\sum }}{\text{X}}_{\text{li}}\;\left(\text{l}\in \text{L}\right)$$

(7) $${\text{M}}_{\text{i}}\leq {\text{U}}_{\text{i}}\;\left(\text{i}\in \text{I}\right)$$

(8) $${\text{M}}_{\text{l}}\leq {\text{U}}_{\text{l}}\;\left(\text{l}\in \text{L}\right)$$

(9) $${\text{C}}_{\text{j}}\geq \overset{\text{I}}{\underset{\text{i}=1}{\sum }}{\text{X}}_{\text{ij}}\;\left(\text{j}\in \text{J}\right)$$

(10) $${\text{C}}_{\text{i}}\geq \overset{\text{L}}{\underset{\text{l}=1}{\sum }}{\text{X}}_{\text{li}}\;\left(\text{i}\in \text{I}\right)$$

(11) $${\text{t}}_{\text{ij}}{\text{X}}_{\text{ij}}\leq {\text{T}}^{\text{max}}{\text{ ​X}}_{\text{ij}}\neq 0,\forall \text{i}\in \text{I,}\forall \text{j}\in \text{J}$$

(12) $${\text{t}}_{\text{li}}{\text{X}}_{\text{lj}}\leq {\text{T}}^{\text{max}}{\text{ ​X}}_{\text{li}}\neq 0,\forall \text{i}\in \text{I,}\forall \text{l}\in \text{L}$$

The objective function shown in Eq. (1) expresses to minimize the total transportation costs of raw materials from suppliers to distribution centers and from distribution centers to factories, respectively. The objective function shown in Eq. (2) aims to minimize the total transportation time from supply centers to distribution centers and from distribution centers to factories, respectively.

Constraints (3) limit the amount of raw material transported from each supplier to each distribution center. Constraints (4) limit the amount of raw material transported from each distribution center to each factory. Constraints (5) illustrate the total amount of raw material entered to each distribution center from supplier centers. Constraints (6) indicate the accumulated amount of raw material sent to the factory by distribution centers. Constraints (7) limit the capacity of raw material entered to each distribution center from supplier centers. Constraints (8) limit the capacity of raw material sent to the factory by distribution centers. Constraints (9) indicate the capacity constraints on each supplier center. Constraints (10) impose the capacity constraints on each distribution center. Constraints (11) and (12) indicate the restriction related to transportation time from each supply center to distribution centers and factories.

## 4 Solution approach

To solve the proposed model, the objective functions (Z_i_) must first be normalized between zero and one to be dimensionless. These objectives are converted to a single function by using formula (13), where Z_1_′ and Z_2_′ are the normalized forms of Z_1_ and Z_2_ objective functions, respectively.

(13) $$\begin{array}{cc} Min: & Z=\theta Z_{1}^{\prime }+ \end{array}\left(1-\theta \right)Z_{2}^{\prime }$$

This function should be reduced to minimize deviations from the ideal. As the Z_1_ has the same importance with Z_2_ in the given model, the value of *θ* is set to 0.5.

### 4.1 Ant colony optimization

The use of swarm intelligence is a relatively new approach to solving problems and inspired from social behavior of insects and other animals. Particularly, there are several methods had been inspired from ant's behavior and there are researches in this field. The most successful method was the versatile optimization method known as ant colony optimization (ACO). Ant colony optimization had been modeled from the behavior of the ant's verities that seek food. These ants remained pheromone and determined the suitable direction for other group members through these materials. In a test called due bridge test, the ant nest is connected to a food source by two bridges with the same length. In this case the ants check around the nest to find the food and finally found a food source by putting a substance called pheromone. Initially, each ant selects one of the paths randomly. Inspire of the existence of random events, the density of pheromone had increased at some points and attracted more ants and resulted in more choosing of that direction by ants. Then Goss et al. (1990) changed the conditions of the experiment and increase the length of one of the bridges and repeated the experiment. In this case, the fluctuations in choosing one path had decreased and the second construction had an important role. Those ants that randomly choose shorter distance, can reach the food source faster and randomly. Thus the shorter path can find pheromone more quickly and this increases the probability of choosing this path by ants. Dorigo and Blum (2005) provided a discussion of theoretical results on ACO. They reviewed some convergence results and then discussed some relations between ACO and other heuristic algorithms for optimization.

The $p_{ij}^{k}$of an k_*th*_ ant transporting from the location i to location j is denoted as follows:

(14) $$\begin{array}{c} \begin{array}{l} p_{ij}^{k}=\left\{ \begin{array}{l} \frac{\tau_{ij}^{\alpha }.\eta_{ij}^{\beta }}{\sum_{l\in N_{i}^{k}}\tau_{il}^{\alpha }.\eta_{il}^{\beta }}\begin{array}{ccc} & \begin{array}{cc} if & j\in N_{i}^{k} \end{array} & \end{array}\\ 0\begin{array}{cccc} & & otherwise & \end{array} \end{array}\right. \end{array} \end{array}$$

where *N*_*i*_^*k*^ expresses the set of locations which k_*th*_ ant must meet at the location *i*. Also, τ_ij_ and *d*_*ij*_ are the pheromone concentration and distance between two location i and location j, where *η*_*ij*_*=1/d*_*ij*_ is the heuristic data. The parameters *α* and *β* denote the relative importance of *τ* and *η* respectively. The pheromone upgrade is done after establishing the routes as follows:

(15) $$\tau_{ij}\leftarrow \left(1-\rho \right).\tau_{ij}+\rho \tau_{0}$$

where ρ is the evaporation rate in (0, 1] that controls the speed of evaporation and τ_0_ is the value of initial pheromone. After ant k makes a tour, the global tour pheromone updating is done as follows:

(16) $$\tau_{ij}\leftarrow \left(1-\rho \right).\tau_{ij}+\varsigma \tau_{0}$$

where ς is the pheromone decay in (0, 1]. This process is repeated and the best solution from all of the iterations is selected as an optimal solution. (Dorigo and Blum, 2005)

### 4.2 Particle Swarm Optimization

Also in this paper, relevant to our problem, a Particle Swarm Optimization (PSO) was developed to solve proposed model. PSO is inspired by the social behavior simulation, was originally designed and developed by Eberhart and Kennedy (1995). It is a population-based swarm intelligence algorithm that was on the basis of the simulation of the social behavior of social organisms such as bird flocking and fish schooling. In the PSO algorithm, the optimization is performed by the set of particles which are communicating with each other. One of the important factors to design a prosperous PSO is to find an appropriate relation between LRP solutions and particles in PSO. Each particle is randomly placed in the d-dimensional space as a candidate solution. Therefore, all particles move in the d-dimensional space of the problem while retrieving historical information collected during the search process. These particles tend to move to better search areas during the search process. Each particle has its location and velocity.

The velocity (*v*_*id*_) and position (*x*_*id*_) updates of the *i*_th_ particle are calculated as follows:

(17) $$v_{id}\left(t+1\right)=w\times v_{d}\left(t\right)+c_{1}\times r_{1}\times \left(pb_{id}\left(t\right)-x_{id}\left(t\right)\right)+c_{2}\times r_{2}\times \left(gb_{id}\left(t\right)-x_{id}\left(t\right)\right)$$

(18) $$x_{id}\left(t+1\right)=x_{id}\left(t\right)+v_{id}\left(t\right)$$

where *r*_1_ and *r*_2_ are two uniformly distributed random numbers in [0,1] and *c*_*1*_ and *c*_*2*_ are the acceleration constants. *v*_*i*_=(*v*_*i1*_*,v*_*i2*_*,...,v*_*id*_) represents the velocity of the *i*_th_ particle, *x*_*i*_*=(x*_*i1*_*,x*_*i2*_*,..., x*_*id*_*)* is the position of the *i*_*th*_ particle, *gb=(gb*_*1*_*,gb*_*2*_*,...,gb*_*D*_*)* is the best position discovered by the whole population and *pb*_*id*_*=(pb*_*i1*_*,pb*_*i2*_*,...,pb*_*id*_*)* is the best previous position yielding the best fitness value for the *i*_th_ particle. In Eq. (18), *w* is the inertia weight used to balance between the local and global search abilities. *t* is the current iteration times, *t*_*max*_ is the total iteration times, *w*_*min*_ is the final inertia weight of the velocity and *w*_max_ is the initial inertia weight of the velocity (Eberhart and Kennedy, 1995).

## 5 Computational results

### 5.1 Parameters tuning

Since the results of all meta-heuristics techniques are sensitive to their parameter setting, extensive simulations are needed to find the appropriate values for the different parameters. Thus the process of inventing a new methodology for solving this problem, allocated by Taguchi (1995). Taguchi added quality and breadth of knowledge through his research in the 1950s and 1960s, which specifically raised the concept of loss cost function. This function integrated scope and diversity with measuring the specification limits. In addition, Taguchi has developed the concept of strength, which means addressing the irregularities in order to ensure the proper functioning of the system (Taguchi, 1995).

In this study, Taguchi method was used in order to determine the appropriate level of each parameter. Based on the structure of the Taguchi method, three levels are first proposed for each of the parameters of the ACO and PSO algorithms. The proposed values are presented in Table 1. Taguchi recommends using the loss function to measure performance characteristics that deviate from the target value. The value of this loss function is further transformed into a signal-to-noise (S/N) ratio. Based on this method, the noise factor descripts the unwanted factors in the evaluated value and the signal value denotes the real value that the system provides.

(19) $$\text{S/N}=-10\text{*log}\left(1\text{/n}\overset{\text{n}}{\underset{\text{i}=1}{\sum }}\text{yi}2\right)$$

where, n = Sample Size, and y = Surface Roughness in that run.

**Table 1:** Parameters and their values for the proposed algorithms.

The ACO parameters			
Level	Pheromone evaporation rate	Population	Iteration
1	0.000001	20	50
2	0.000003	30	100
3	0.000006	40	200

The PSO parameters				
Level	Individual intelligence rate	Social intelligence rate	Population	Iteration
1	3	1	20	50
2	4.5	2	30	100
3	6	3	40	200

**Figure 1:**
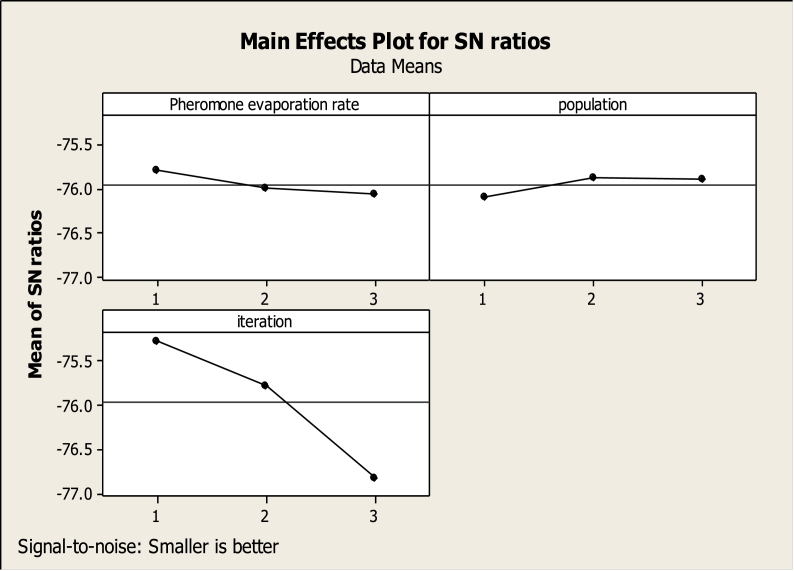
Main effects plot for the S/N ratio of ACO.

**Figure 2:**
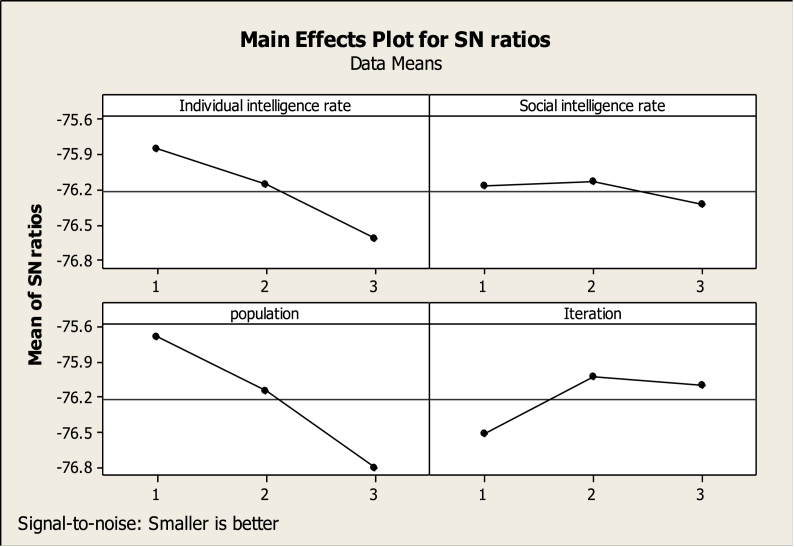
Main effects plot for the S/N ratio of PSO.

According to the data in Table 1, the target (location-routing of perishable material supply) sizes small, medium and large, has been performed three times. Referring to Taguchi standard table (Taguchi, 1995), for the L_9_ (32) Taguchi scheme, the ACO and PSO algorithms were executed for the following scenarios with Minitab (16) software, the S/N charts are presented as Figures 1 and 2.

According to Eq. (19), the suitable value for each parameter is the lowest S/N value. Therefore, the parameter set obtained through optimization for ACO & PSO algorithms are presented in Table 2.

**Table 2:** Parameter set obtained through optimization for ACO & PSO algorithms.

The ACO parameters		
Pheromone evaporation rate	Population	Iteration
0.000006	20	200

The PSO parameters			
Individual intelligence rate	Social intelligence rate	Population	Iteration
6	3	40	50

### 5.2 Numerical calculations

To prove the feasibility of proposed model and validity of the proposed algorithms, a hypothetical example of randomize generated data about the dairy industry is expressed in this section. Tables 3 and 4 show the distances between 13 potential locations for suppliers, 4 potential locations for distribution centers and 2 potential locations for factories. The distance unit is kilometer and the maximum time for each path is 30 min.

**Table 3:** Distance between potential locations of suppliers & distribution centers in kilometer.

D_ij_	J_1_	J_2_	J_3_	J_4_	J_5_	J_6_	J_7_	J_8_	J_9_	J_10_	J_11_	J_12_	J_13_
I_1_	2	5	10	25	15	41	35	35	40	50	60	70	100
I_2_	40	41	25	15	25	10	25	10	32	20	35	35	40
I_3_	32	20	32	30	15	25	2	5	10	15	20	25	25
I_4_	100	70	70	80	60	70	25	31	20	35	5	10	15

**Table 4:** Distance between potential locations of distribution centers & factories in kilometer.

D_li_	I_1_	I_2_	I_3_	I_4_
L_1_	10	10	25	70
L_2_	70	30	20	10

Tables 5 and 6 represent the supply capacity of the perishable raw material by suppliers and distribution centers, respectively. The capacity unit is the kilogram to measure quantity of raw material.

**Table 5:** The capacity of supplier centers in kilogram.

	J_1_	J_2_	J_3_	J_4_	J_5_	J_6_	J_7_	J_8_	J_9_	J_10_	J_11_	J_12_	J_13_
C_j_	50	100	20	30	80	90	200	50	150	90	200	70	140

**Table 6:** The capacity of distribution centers in kilogram.

	I_1_	I_2_	I_3_	I_4_
C_i_	300	350	500	250

We ran all computational experiments on a Linux-based workstation with a 2.4 GHz processor and 2 GB RAM. A summary of computational results is reported in the following table. Tables 7 and 8, respectively, show the results of the ACO and PSO algorithms, that specify which supplier should be sent perishable raw material to which distribution center. Also, these tables show the volume of perishable raw materials sent from suppliers to distribution centers. Tables 9 and 10, respectively, show the results of the ACO and PSO algorithms that specify which distribution center should be sent perishable raw material to which factory. Also, these tables show the volume of perishable raw materials sent from distribution centers to factories.

**Table 7:** The ACO results of the volume transferred from suppliers to distribution centers in kilogram.

	J_1_	J_2_	J_3_	J_4_	J_5_	J_6_	J_7_	J_8_	J_9_	J_10_	J_11_	J_12_	J_13_
I_1_	50	100	20	0	80	0	0	0	0	0	0	0	0
I_2_	0	0	0	30	0	90	50	50	0	90	0	0	0
I_3_	0	0	0	0	0	0	150	0	150	0	50	0	110
I_4_	0	0	0	0	0	0	0	0	0	0	150	70	30

**Table 8:** The PSO results of the volume transferred from suppliers to distribution centers in kilogram.

	J_1_	J_2_	J_3_	J_4_	J_5_	J_6_	J_7_	J_8_	J_9_	J_10_	J_11_	J_12_	J_13_
I_1_	0	80	20	0	60	0	50	0	0	0	0	0	0
I_2_	50	0	0	30	0	90	0	50	0	75	0	0	0
I_3_	0	20	0	0	0	0	150	0	85	0	50	0	100
I_4_	0	0	0	0	20	0	0	0	65	15	150	70	40

**Table 9:** The ACO results of volume transferred from distribution centers to factories in kilogram.

	I_1_	I_2_	I_3_	I_4_
L_1_	250	310	0	0
L_2_	0	0	460	250

**Table 10:** The PSO results of volume transferred from distribution centers to factories in kilogram.

	I_1_	I_2_	I_3_	I_4_
L_1_	200	280	0	30
L_2_	50	30	460	220

**Figure 3:**
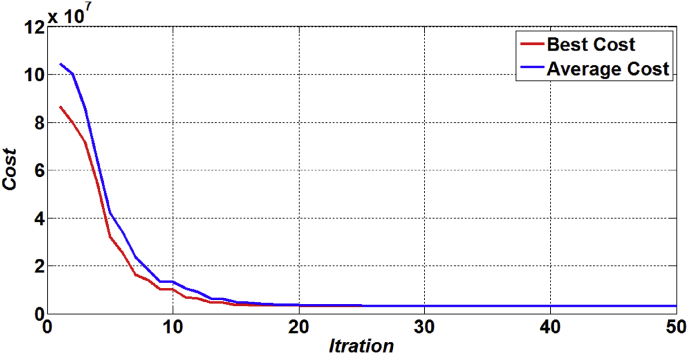
The best and average fitness of ACO.

**Figure 4:**
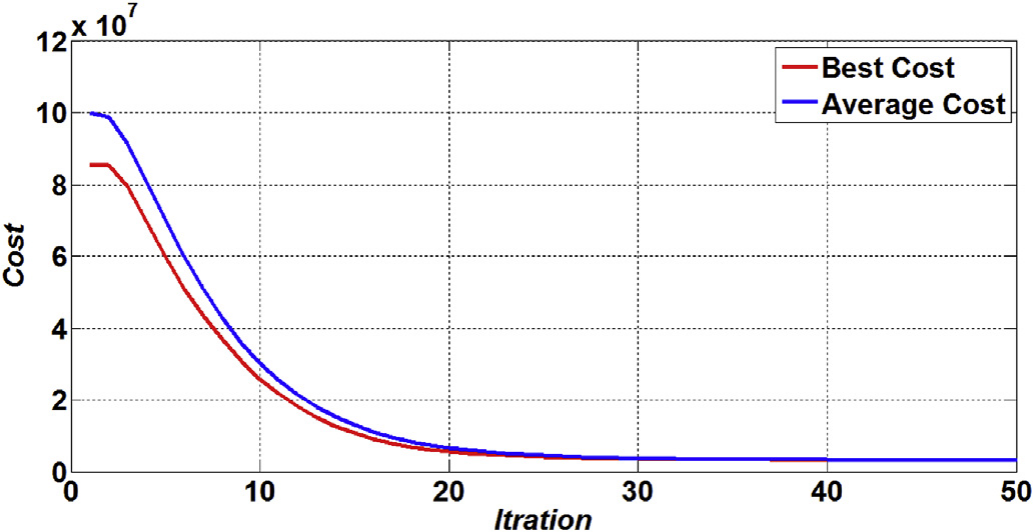
The best and average fitness of PSO.

Figure 3 and 4 show the average and the best fitness curves for the ACO and PSO algorithms, respectively. As can be seen from these figures, it can be said that the ACO is greater than PSO on average and the best fitness rate in iterations.

**Figure 5:**
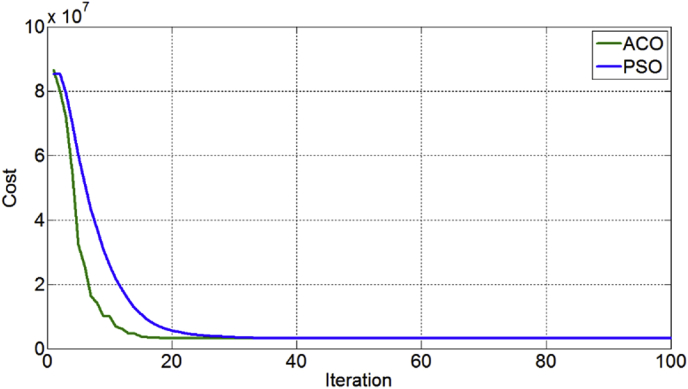
The compare convergence curve between ACO and PSO algorithms.

Moreover, in order to compare performance of the ACO and PSO algorithms to solve the proposed model and to validate the robustness of the proposed algorithms, convergence curves of the ACO and PSO is compared in Figure 5. As is shown in this figure, in convergence metric, the results of the performance measures show that the ACO has better convergence compared to the PSO. On the other hand, it can be said that that the ACO is greater than PSO in speed convergence rate and the number of solutions iterations.

## 6 Conclusions and recommendations

Managers recognize that there is a strong relation between the location of facilities, the allocation of suppliers, vehicles and customers to the facilities and in the design of routes around the facilities. The number and location of facilities, fleet size and the path structure are determined regarding locations and characteristics of suppliers and customers. The aim of this paper was to investigate the ordering planning of a supply chain with multi supplier, multi distribution center, multi customer and one perishable raw material. This paper provided a mathematical model taking in consideration the limitation on raw material corruptibility's (perishable material). The most important goals of the proposed model are to minimize transportation costs and transport time of perishable raw materials between suppliers, distribution centers and factory with considering the capacity of them. Considering the proposed model belongs to the category of NP-hard combinatorial optimization problems, the Ant Colony Optimization algorithm and Particle Swarm Optimization algorithm were employed to solve it. Also, In order to improve performances of ACO and PSO parameters, a Taguchi experimental design method was applied to set their proper values. To prove the feasibility of proposed model and validity of the proposed algorithms, a hypothetical example of randomize generated data about the dairy industry was expressed in this paper. The numerical results of the suggested algorithms and the proposed model were analyzed. In order to assess the reliability of the solution, the results of the proposed algorithms (ACO and PSO) were compared with each other. In convergence metric, the results of the performance measures show that the ACO has better convergence compared to the PSO. On the other hand, it can be said that that the ACO is greater than PSO in speed convergence rate and the number of solutions iterations.

The following items are recommended for Future researches:

• Developing the proposed model for multi-product problems
• Developing the proposed model with considering risk in transportation
• Developing the proposed model in multi periods of time
• Developing the proposed model with considering heterogeneous capacities for platforms
• Developing the proposed model with probabilistic parameters for demand and costs
• Increasing the shelf life of raw material by special equipment
• Designing other meta-heuristic algorithms and comparing results

## Declarations

### Author contribution statement

Ali Yaghoubi: Conceived and designed the experiments; Performed the experiments; Analyzed and interpreted the data; Contributed reagents, materials, analysis tools or data; Wrote the paper.

Farideh Akrami: Contributed reagents, materials, analysis tools or data; Wrote the paper.

### Funding statement

This research did not receive any specific grant from funding agencies in the public, commercial, or not-for-profit sectors.

### Competing interest statement

The authors declare no conflict of interest.

### Additional information

No additional information is available for this paper.
